# Tetra-μ_2_-oxido-di-μ_4_-peroxido-tetra­kis[diphenyl­anti­mony(V)] chloro­form disolvate

**DOI:** 10.1107/S160053680804419X

**Published:** 2009-02-06

**Authors:** Richard Betz, Christoph Lindner, Peter Klüfers, Peter Mayer

**Affiliations:** a Ludwig-Maximilians Universität, Department Chemie und Biochemie, Butenandtstrasse 5–13 (Haus D), 81377 München, Germany

## Abstract

The title compound, [Sb_4_(C_6_H_5_)_8_O_4_(O_2_)_2_]·2CHCl_3_, contains a tetranuclear antimony(V) core, bridged by oxide and peroxide ligands. Two cores form centrosymmetric dimers by intermolecular C—H⋯O contacts. These dimeric units are further connected by chloro­form solvent mol­ecules involved in C—H⋯O and C—H⋯Cl inter­actions into strands along [010]. The five-membered Sb_2_O_3_ rings in the Sb_4_O_8_ core invariably adopt envelope conformations.

## Related literature

The title compound was unintentionally obtained upon the attempted synthesis of the cyclo­butane­carboxylic acid addition compound derived from triphen­ylstibane oxide. For preparation of carboxyl­ate derivatives of triphen­ylstibane oxide, see: Domagala *et al.* (1989 ▶, 1990 ▶). For the crystal structure of the solvent-free tetra­nuclear cluster, see: Breunig *et al.* (2002 ▶); Sharutin *et al.* (2004 ▶). For details on graph-set analysis of hydrogen bonds, see: Etter *et al.* (1990 ▶); Bernstein *et al.* (1995 ▶). For details on puckering analysis, see: Cremer & Pople (1975 ▶).
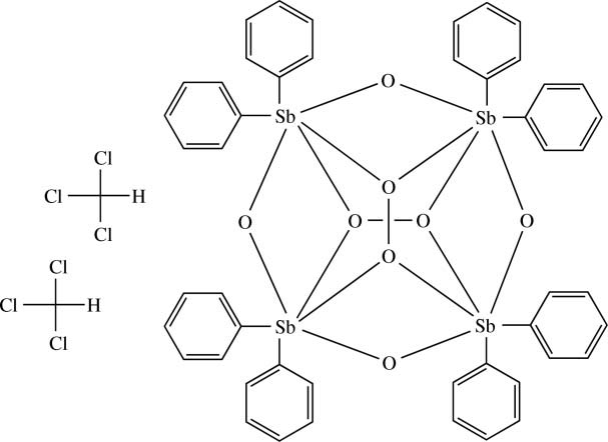


## Experimental

### 

#### Crystal data

[Sb_4_(C_6_H_5_)_8_O_4_(O_2_)_2_]·2CHCl_3_
*M* *_r_* = 1470.54
Monoclinic, 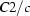

*a* = 23.0764 (3) Å
*b* = 13.7353 (2) Å
*c* = 34.6597 (4) Å
β = 105.3795 (6)°
*V* = 10592.4 (2) Å^3^
*Z* = 8
Mo *K*α radiation
μ = 2.37 mm^−1^
*T* = 200 (2) K
0.21 × 0.19 × 0.16 mm

#### Data collection

Nonius KappaCCD diffractometer
Absorption correction: multi-scan (*SADABS*; Sheldrick, 2001 ▶) *T*_min_ = 0.599, *T*_max_ = 0.684
71535 measured reflections
12127 independent reflections
8489 reflections with *I* > 2σ(*I*)
*R*_int_ = 0.059

#### Refinement

*R*[*F* ^2^ > 2σ(*F* ^2^)] = 0.037
*wR*(*F* ^2^) = 0.082
*S* = 1.04
12127 reflections
614 parameters
H-atom parameters constrained
Δρ_max_ = 0.98 e Å^−3^
Δρ_min_ = −0.89 e Å^−3^

### 

Data collection: *COLLECT* (Nonius, 2004 ▶); cell refinement: *SCALEPACK* (Otwinowski & Minor, 1997 ▶); data reduction: *DENZO* (Otwinowski & Minor, 1997 ▶) and *SCALEPACK*; program(s) used to solve structure: *SIR97* (Altomare *et al.*, 1999 ▶); program(s) used to refine structure: *SHELXL97* (Sheldrick, 2008 ▶); molecular graphics: *ORTEP-3* (Farrugia, 1997 ▶) and *Mercury* (Macrae *et al.*, 2006 ▶); software used to prepare material for publication: *SHELXL97* and *PLATON* (Spek, 2003 ▶).

## Supplementary Material

Crystal structure: contains datablocks global, I. DOI: 10.1107/S160053680804419X/gk2183sup1.cif

Structure factors: contains datablocks I. DOI: 10.1107/S160053680804419X/gk2183Isup2.hkl

Additional supplementary materials:  crystallographic information; 3D view; checkCIF report

## Figures and Tables

**Table 1:** Hydrogen-bond geometry (Å, °)

*D*—H⋯*A*	*D*—H	H⋯*A*	*D*⋯*A*	*D*—H⋯*A*
C74—H74⋯Cl95^i^	0.95	2.81	3.345 (6)	117
C98—H98⋯O231^i^	1.00	2.29	3.229 (6)	156
C99—H99⋯O122^ii^	1.00	2.27	3.275 (6)	177
C99—H99⋯O342^ii^	1.00	2.46	3.343 (6)	147
C23—H23⋯O412^iii^	0.95	2.60	3.482 (6)	154

Symmetry codes: (i) 
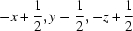
; (ii) 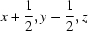; (iii) 
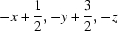
.

**Fig. 1.**
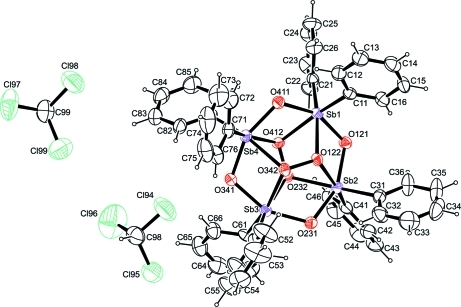
The molecular structure of the title compound, with atom labels and anisotropic displacement ellipsoids drawn at the 50% probability level.

**Fig. 2.**
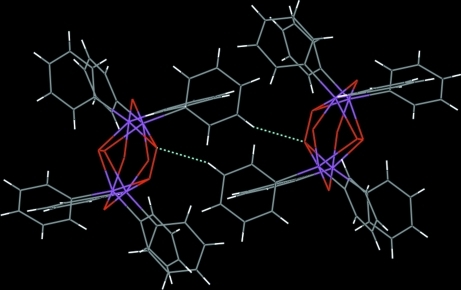
Centrosymmetric dimers in the crystal structure of the title compound, formed by H23···O412 contacts (cf. Table 1).

**Fig. 3.**
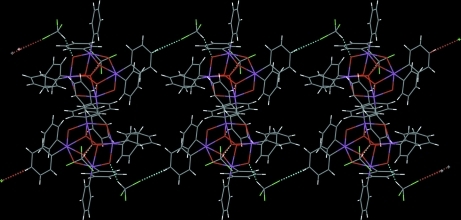
Infinite strands formed in the crystal structure of the title compound, viewed along [1 0 0]. For details about contacts cf. Table 1.

**Fig. 4.**
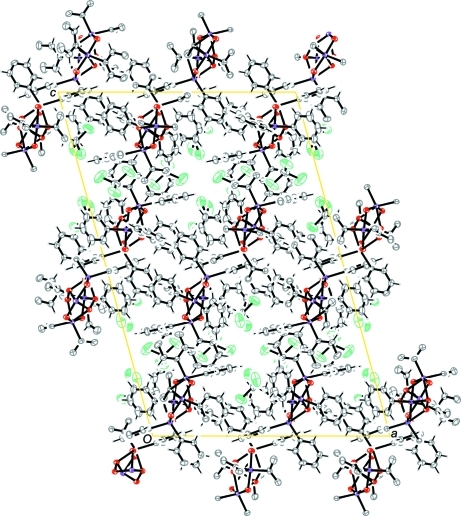
The packing of the title compound, viewed along [0 1 0].

## supplementary crystallographic information

### Comment

For a comprehensive study about the coordination behaviour of main group elements in their highest oxidation states toward carboxylato ligands, the synthesis of a derivative of antimony(V) was attempted. Instead of the expected mononuclear reaction product, a tetranuclear cluster was obtained.

In the molecule two bridging peroxido-groups and four oxido bridges connect the four Sb atoms in terms of annealed four-, five- and six-membered rings (Fig 1). According to a puckering analysis (Cremer & Pople, 1975), the five-membered rings invariably adopt *envelope* conformations:

Sb1–O121–Sb2–O232–O412 *E*_O121_ (*Q_2_* = 0.720 (2) Å, *φ_2_* = 36.53 (18)°), Sb1–O122–Sb2–O232–O412 ^O122^*E* (*Q_2_* = 1.205 (2) Å, *φ_2_* = 215.88 (11)°), Sb1–O122–O342–Sb4–O411 ^O411^*E* (*Q_2_* = 0.696 (2) Å, *φ_2_* = 145.08 (19)°), Sb1–O122–O342–Sb4–O412 *E*_O412_ (*Q_2_* = 1.197 (2) Å, *φ_2_* = 323.61 (11)°), Sb2–O122–O342–Sb3–O231 *E*_O231_ (*Q_2_* = 0.740 (2) Å, *φ_2_* = 324.46 (18)°), Sb2–O122–O342–Sb3–O232 ^O232^*E* (*Q_2_* = 1.190 (2) Å, *φ_2_* = 143.72 (11)°), Sb3–O232–O412–Sb4–O341 ^341^*E* (*Q_2_* = 0.658 (2) Å, *φ_2_* = 144.0 (2)°), Sb3–O232–O412–Sb4–O342 *E*_O342_ (*Q_2_* = 1.210 (2) Å, *φ_2_* = 323.96 (11)°).

Both peroxido groups together with two diametrically arranged Sb atoms are part of six-membered rings each which adopt *twist-boat* conformations. For Sb1–O122–O342–Sb3–O232–O412, the respective puckering parameters were found to be *Q* = 1.039 (3) Å, *θ* = 89.80 (11)° and *φ* = 89.61 (10)°. For Sb2–O122–O342–Sb4–O412–O232, the respective puckering parameters were found to be *Q* = 1.039 (3) Å, *θ* = 90.08 (11)° and *φ* = 270.20 (10)°.

In the crystal structure, two clusters form centrosymmetric dimers by weak C–H···O contacts stemming from an H atom in *meta* positon of one of the aromatic moieties and one of the peroxido O atoms (Fig. 2). The two solvent molecules do not show an identical behaviour in the crystal structure. While the first serves as a linker for the dimeric units upon the formation of C–H···Cl contacts as well as C–H···O contacts furnishing strands along [0 1 0] (Fig. 3), the second only participates in a finite, bifurcated C–H···O hydrogen bond (*cf.* Table 1). In terms of graph-set analysis, the unitary descriptor of these contacts is *DDDDR*^2^_2_(12). A binary descriptor is *C*^2^_2_(12).

The packing of the compound is shown in Figure 4.

### Experimental

The compound was unintentionally prepared in adoption of a published procedure (Domagala *et al.*, 1989; Domagala *et al.*, 1990) aimed at the synthesis of carboxylato derivatives of triphenylstibane oxide upon the reaction of cyclobutanecarboxylic acid and triphenylstibane oxide in chloroform. Crystals suitable for X-ray analysis were obtained upon free evaporation of the solvent.

### Refinement

Carbon-bound H-atoms were placed in calculated positions (C—H 0.95 Å for aromatic C atoms, C—H 1.00 Å for methine groups) and were included in the refinement in the riding model approximation, with *U*(H) set to 1.2*U*_eq_(C).

### Crystal data

**Table:**

[Sb_4_(C_6_H_5_)_8_O_4_(O_2_)_2_]·2CHCl_3_	*F*(000) = 5696
*M**_r_* = 1470.54	*D*_x_ = 1.844 Mg m^−^^3^
Monoclinic, *C*2/*c*	Mo *K*α radiation, λ = 0.71073 Å
Hall symbol: -C 2yc	Cell parameters from 79731 reflections
*a* = 23.0764 (3) Å	θ = 3.1–27.5°
*b* = 13.7353 (2) Å	µ = 2.37 mm^−^^1^
*c* = 34.6597 (4) Å	*T* = 200 K
β = 105.3795 (6)°	Block, colourless
*V* = 10592.4 (2) Å^3^	0.21 × 0.19 × 0.16 mm
*Z* = 8	

### Data collection

**Table:**

Nonius KappaCCD diffractometer	12127 independent reflections
Radiation source: rotating anode	8489 reflections with *I* > 2σ(*I*)
MONTEL, graded multilayered X-ray optics	*R*_int_ = 0.059
φ and ω scans	θ_max_ = 27.5°, θ_min_ = 3.2°
Absorption correction: multi-scan (*SADABS*; Sheldrick, 2001)	*h* = −29→29
*T*_min_ = 0.599, *T*_max_ = 0.684	*k* = −17→17
71535 measured reflections	*l* = −45→45

### Refinement

**Table:**

Refinement on *F*^2^	Primary atom site location: structure-invariant direct methods
Least-squares matrix: full	Secondary atom site location: difference Fourier map
*R*[*F*^2^ > 2σ(*F*^2^)] = 0.037	Hydrogen site location: inferred from neighbouring sites
*wR*(*F*^2^) = 0.082	H-atom parameters constrained
*S* = 1.04	*w* = 1/[σ^2^(*F*_o_^2^) + (0.0299*P*)^2^ + 28.2696*P*] where *P* = (*F*_o_^2^ + 2*F*_c_^2^)/3
12127 reflections	(Δ/σ)_max_ = 0.001
614 parameters	Δρ_max_ = 0.98 e Å^−^^3^
0 restraints	Δρ_min_ = −0.89 e Å^−^^3^

### Fractional atomic coordinates and isotropic or equivalent isotropic displacement parameters (Å^2^)

**Table:**

	*x*	*y*	*z*	*U*_iso_*/*U*_eq_	
Sb1	0.088803 (12)	0.71783 (2)	0.039333 (8)	0.02610 (7)	
Sb2	0.127447 (13)	0.902037 (19)	0.099132 (8)	0.02731 (8)	
Sb3	0.207389 (12)	0.75923 (2)	0.167703 (8)	0.02710 (8)	
Sb4	0.167522 (12)	0.57351 (2)	0.107803 (8)	0.02710 (8)	
Cl94	0.29511 (10)	0.49729 (15)	0.23138 (6)	0.1234 (9)	
Cl95	0.37828 (10)	0.60400 (13)	0.29061 (6)	0.1083 (8)	
Cl96	0.42058 (14)	0.4612 (2)	0.24610 (8)	0.1591 (11)	
Cl97	0.50002 (9)	0.06841 (13)	0.16428 (8)	0.1175 (8)	
Cl98	0.41456 (10)	0.1776 (2)	0.10677 (6)	0.1289 (9)	
Cl99	0.47561 (9)	0.26532 (14)	0.18030 (6)	0.0968 (6)	
O121	0.10064 (12)	0.85900 (19)	0.04311 (8)	0.0296 (7)	
O122	0.08436 (12)	0.75740 (18)	0.10162 (8)	0.0270 (6)	
O231	0.16180 (12)	0.88177 (19)	0.15700 (8)	0.0299 (7)	
O232	0.20051 (12)	0.79408 (18)	0.10372 (7)	0.0259 (6)	
O341	0.22559 (12)	0.62582 (19)	0.15499 (8)	0.0306 (7)	
O342	0.12076 (12)	0.69245 (19)	0.13249 (8)	0.0270 (6)	
O411	0.10091 (12)	0.58347 (19)	0.05934 (8)	0.0296 (7)	
O412	0.18258 (12)	0.71073 (18)	0.07678 (7)	0.0259 (6)	
C11	−0.00614 (19)	0.7104 (3)	0.02102 (12)	0.0296 (10)	
C12	−0.0348 (2)	0.6211 (3)	0.01796 (14)	0.0412 (12)	
H12	−0.0119	0.5635	0.0257	0.049*	
C13	−0.0970 (2)	0.6153 (4)	0.00367 (15)	0.0470 (13)	
H13	−0.1164	0.5537	0.0013	0.056*	
C14	−0.1302 (2)	0.6981 (4)	−0.00691 (14)	0.0468 (13)	
H14	−0.1728	0.6941	−0.0163	0.056*	
C15	−0.1021 (2)	0.7878 (4)	−0.00396 (14)	0.0449 (12)	
H15	−0.1254	0.8451	−0.0114	0.054*	
C16	−0.0401 (2)	0.7943 (3)	0.00986 (13)	0.0365 (11)	
H16	−0.0209	0.8559	0.0117	0.044*	
C21	0.11666 (18)	0.6999 (3)	−0.01363 (11)	0.0280 (9)	
C22	0.1652 (2)	0.7500 (4)	−0.01982 (13)	0.0405 (12)	
H22	0.1876	0.7926	0.0002	0.049*	
C23	0.1815 (2)	0.7382 (4)	−0.05526 (15)	0.0475 (13)	
H23	0.2147	0.7733	−0.0595	0.057*	
C24	0.1498 (2)	0.6760 (4)	−0.08433 (14)	0.0493 (13)	
H24	0.1614	0.6674	−0.1084	0.059*	
C25	0.1012 (2)	0.6263 (4)	−0.07828 (14)	0.0507 (13)	
H25	0.0790	0.5836	−0.0984	0.061*	
C26	0.0844 (2)	0.6383 (3)	−0.04323 (13)	0.0414 (12)	
H26	0.0507	0.6040	−0.0394	0.050*	
C31	0.04830 (19)	0.9749 (3)	0.10167 (13)	0.0330 (10)	
C32	0.0258 (2)	0.9705 (4)	0.13449 (15)	0.0508 (13)	
H32	0.0452	0.9312	0.1567	0.061*	
C33	−0.0254 (3)	1.0235 (5)	0.1354 (2)	0.0733 (19)	
H33	−0.0408	1.0198	0.1582	0.088*	
C34	−0.0531 (3)	1.0804 (4)	0.1039 (2)	0.073 (2)	
H34	−0.0875	1.1177	0.1048	0.088*	
C35	−0.0315 (3)	1.0839 (4)	0.0709 (2)	0.0731 (19)	
H35	−0.0513	1.1225	0.0486	0.088*	
C36	0.0187 (2)	1.0317 (4)	0.07003 (17)	0.0563 (15)	
H36	0.0333	1.0348	0.0469	0.068*	
C41	0.1852 (2)	1.0153 (3)	0.09013 (13)	0.0360 (11)	
C42	0.1767 (2)	1.1089 (3)	0.10279 (13)	0.0420 (12)	
H42	0.1461	1.1215	0.1158	0.050*	
C43	0.2134 (3)	1.1841 (4)	0.09620 (15)	0.0534 (15)	
H43	0.2075	1.2486	0.1044	0.064*	
C44	0.2579 (3)	1.1651 (4)	0.07793 (17)	0.0578 (16)	
H44	0.2829	1.2165	0.0735	0.069*	
C45	0.2666 (2)	1.0719 (4)	0.06597 (17)	0.0590 (15)	
H45	0.2981	1.0593	0.0537	0.071*	
C46	0.2299 (2)	0.9963 (4)	0.07174 (15)	0.0460 (12)	
H46	0.2357	0.9322	0.0631	0.055*	
C51	0.1913 (2)	0.7331 (3)	0.22389 (12)	0.0328 (10)	
C52	0.1375 (3)	0.7048 (5)	0.22814 (16)	0.0693 (18)	
H52	0.1048	0.6971	0.2050	0.083*	
C53	0.1288 (3)	0.6866 (5)	0.26579 (18)	0.083 (2)	
H53	0.0908	0.6650	0.2682	0.099*	
C54	0.1752 (3)	0.7000 (4)	0.29903 (16)	0.0643 (17)	
H54	0.1694	0.6897	0.3249	0.077*	
C55	0.2293 (3)	0.7280 (5)	0.29511 (16)	0.0740 (19)	
H55	0.2619	0.7362	0.3183	0.089*	
C56	0.2381 (2)	0.7450 (4)	0.25760 (14)	0.0573 (15)	
H56	0.2764	0.7648	0.2552	0.069*	
C61	0.29731 (19)	0.8100 (3)	0.18132 (11)	0.0316 (10)	
C62	0.3107 (2)	0.9081 (3)	0.18207 (15)	0.0466 (13)	
H62	0.2794	0.9552	0.1772	0.056*	
C63	0.3703 (2)	0.9371 (4)	0.18996 (17)	0.0594 (15)	
H63	0.3797	1.0045	0.1903	0.071*	
C64	0.4154 (2)	0.8702 (5)	0.19728 (16)	0.0592 (15)	
H64	0.4560	0.8911	0.2029	0.071*	
C65	0.4023 (2)	0.7729 (4)	0.19659 (17)	0.0574 (15)	
H65	0.4339	0.7264	0.2018	0.069*	
C66	0.3434 (2)	0.7420 (4)	0.18832 (15)	0.0461 (13)	
H66	0.3344	0.6745	0.1874	0.055*	
C71	0.12815 (19)	0.4632 (3)	0.13515 (13)	0.0338 (10)	
C72	0.0901 (2)	0.3974 (3)	0.11120 (17)	0.0506 (13)	
H72	0.0822	0.4018	0.0829	0.061*	
C73	0.0633 (3)	0.3247 (4)	0.1281 (2)	0.0724 (19)	
H73	0.0378	0.2784	0.1115	0.087*	
C74	0.0736 (3)	0.3196 (4)	0.1683 (2)	0.072 (2)	
H74	0.0541	0.2709	0.1798	0.087*	
C75	0.1116 (3)	0.3834 (5)	0.19267 (18)	0.0660 (17)	
H75	0.1193	0.3782	0.2209	0.079*	
C76	0.1390 (2)	0.4560 (4)	0.17595 (14)	0.0449 (12)	
H76	0.1654	0.5008	0.1928	0.054*	
C81	0.23468 (18)	0.5023 (3)	0.08724 (13)	0.0298 (10)	
C82	0.2754 (2)	0.4478 (3)	0.11550 (14)	0.0407 (11)	
H82	0.2696	0.4403	0.1415	0.049*	
C83	0.3241 (2)	0.4043 (4)	0.10675 (16)	0.0507 (14)	
H83	0.3514	0.3665	0.1264	0.061*	
C84	0.3329 (2)	0.4159 (4)	0.06942 (17)	0.0526 (14)	
H84	0.3668	0.3871	0.0633	0.063*	
C85	0.2925 (2)	0.4692 (4)	0.04080 (16)	0.0568 (15)	
H85	0.2984	0.4761	0.0148	0.068*	
C86	0.2435 (2)	0.5128 (3)	0.04968 (14)	0.0437 (12)	
H86	0.2160	0.5499	0.0299	0.052*	
C98	0.3629 (3)	0.4924 (4)	0.26670 (16)	0.0632 (16)	
H98	0.3603	0.4421	0.2870	0.076*	
C99	0.4816 (2)	0.1812 (4)	0.14399 (18)	0.0631 (16)	
H99	0.5141	0.2033	0.1318	0.076*	

### Atomic displacement parameters (Å^2^)

**Table:**

	*U*^11^	*U*^22^	*U*^33^	*U*^12^	*U*^13^	*U*^23^
Sb1	0.02608 (15)	0.02778 (15)	0.02427 (14)	−0.00118 (12)	0.00635 (12)	0.00146 (12)
Sb2	0.03027 (16)	0.02492 (15)	0.02629 (15)	−0.00048 (12)	0.00672 (12)	0.00207 (12)
Sb3	0.02836 (16)	0.02936 (15)	0.02335 (14)	0.00041 (12)	0.00646 (12)	0.00145 (12)
Sb4	0.02789 (16)	0.02582 (15)	0.02756 (15)	0.00039 (12)	0.00731 (12)	0.00218 (12)
Cl94	0.1175 (17)	0.0941 (14)	0.1124 (16)	−0.0115 (13)	−0.0506 (14)	0.0128 (12)
Cl95	0.1366 (18)	0.0613 (11)	0.0967 (14)	−0.0019 (11)	−0.0223 (13)	0.0020 (10)
Cl96	0.180 (3)	0.183 (3)	0.152 (2)	0.044 (2)	0.110 (2)	0.030 (2)
Cl97	0.0737 (13)	0.0612 (11)	0.208 (2)	0.0023 (9)	0.0206 (14)	0.0333 (13)
Cl98	0.0982 (16)	0.180 (2)	0.0874 (14)	0.0505 (16)	−0.0120 (12)	−0.0320 (15)
Cl99	0.0889 (13)	0.0915 (13)	0.1128 (15)	−0.0045 (11)	0.0315 (12)	−0.0251 (12)
O121	0.0348 (17)	0.0282 (15)	0.0239 (14)	−0.0009 (13)	0.0046 (13)	0.0023 (12)
O122	0.0288 (15)	0.0262 (15)	0.0245 (15)	0.0018 (12)	0.0045 (12)	0.0065 (12)
O231	0.0348 (17)	0.0301 (15)	0.0250 (14)	0.0044 (13)	0.0083 (13)	−0.0010 (12)
O232	0.0273 (15)	0.0256 (14)	0.0248 (14)	−0.0030 (12)	0.0069 (12)	−0.0007 (12)
O341	0.0316 (16)	0.0308 (16)	0.0261 (15)	0.0036 (13)	0.0016 (13)	0.0013 (12)
O342	0.0285 (15)	0.0287 (15)	0.0242 (14)	0.0018 (12)	0.0075 (12)	0.0071 (12)
O411	0.0307 (16)	0.0258 (15)	0.0311 (15)	−0.0004 (13)	0.0061 (13)	0.0011 (12)
O412	0.0310 (16)	0.0240 (14)	0.0228 (14)	−0.0013 (12)	0.0074 (12)	−0.0029 (12)
C11	0.030 (2)	0.034 (2)	0.025 (2)	−0.0013 (19)	0.0076 (18)	−0.0006 (19)
C12	0.037 (3)	0.037 (3)	0.048 (3)	−0.002 (2)	0.008 (2)	−0.001 (2)
C13	0.037 (3)	0.046 (3)	0.055 (3)	−0.008 (2)	0.007 (2)	−0.005 (3)
C14	0.031 (3)	0.063 (3)	0.044 (3)	−0.002 (3)	0.006 (2)	−0.004 (3)
C15	0.036 (3)	0.051 (3)	0.042 (3)	0.013 (2)	0.000 (2)	0.005 (2)
C16	0.038 (3)	0.037 (3)	0.034 (2)	−0.003 (2)	0.008 (2)	0.006 (2)
C21	0.029 (2)	0.032 (2)	0.023 (2)	0.0038 (19)	0.0072 (18)	0.0032 (18)
C22	0.033 (3)	0.056 (3)	0.034 (3)	−0.005 (2)	0.010 (2)	−0.005 (2)
C23	0.035 (3)	0.064 (3)	0.046 (3)	−0.003 (3)	0.017 (2)	0.004 (3)
C24	0.054 (3)	0.063 (3)	0.036 (3)	0.012 (3)	0.021 (2)	0.002 (3)
C25	0.061 (4)	0.059 (3)	0.032 (3)	−0.003 (3)	0.012 (2)	−0.013 (2)
C26	0.043 (3)	0.043 (3)	0.040 (3)	−0.007 (2)	0.014 (2)	−0.004 (2)
C31	0.027 (2)	0.030 (2)	0.041 (3)	−0.0007 (19)	0.007 (2)	−0.002 (2)
C32	0.038 (3)	0.066 (4)	0.046 (3)	0.005 (3)	0.007 (2)	−0.003 (3)
C33	0.040 (3)	0.102 (5)	0.080 (4)	0.003 (4)	0.021 (3)	−0.023 (4)
C34	0.038 (3)	0.053 (4)	0.124 (6)	0.014 (3)	0.016 (4)	−0.010 (4)
C35	0.051 (4)	0.051 (4)	0.114 (6)	0.020 (3)	0.017 (4)	0.033 (4)
C36	0.050 (3)	0.050 (3)	0.071 (4)	0.019 (3)	0.021 (3)	0.025 (3)
C41	0.036 (3)	0.035 (3)	0.030 (2)	−0.005 (2)	−0.003 (2)	0.005 (2)
C42	0.051 (3)	0.037 (3)	0.036 (3)	−0.006 (2)	0.007 (2)	0.001 (2)
C43	0.063 (4)	0.033 (3)	0.050 (3)	−0.018 (3)	−0.009 (3)	0.004 (2)
C44	0.051 (4)	0.049 (3)	0.065 (4)	−0.022 (3)	0.001 (3)	0.016 (3)
C45	0.046 (3)	0.060 (4)	0.076 (4)	−0.014 (3)	0.024 (3)	0.009 (3)
C46	0.041 (3)	0.039 (3)	0.059 (3)	−0.003 (2)	0.015 (3)	0.000 (2)
C51	0.036 (3)	0.037 (2)	0.027 (2)	0.000 (2)	0.011 (2)	0.0013 (19)
C52	0.052 (4)	0.123 (6)	0.037 (3)	−0.019 (4)	0.019 (3)	0.002 (3)
C53	0.071 (4)	0.135 (6)	0.056 (4)	−0.032 (4)	0.040 (3)	−0.001 (4)
C54	0.097 (5)	0.067 (4)	0.037 (3)	−0.007 (4)	0.031 (3)	0.007 (3)
C55	0.076 (5)	0.112 (5)	0.030 (3)	−0.001 (4)	0.007 (3)	0.008 (3)
C56	0.044 (3)	0.093 (4)	0.035 (3)	−0.001 (3)	0.011 (2)	0.008 (3)
C61	0.030 (2)	0.043 (3)	0.020 (2)	−0.004 (2)	0.0040 (18)	0.0016 (19)
C62	0.041 (3)	0.040 (3)	0.058 (3)	−0.007 (2)	0.012 (2)	−0.006 (2)
C63	0.053 (4)	0.046 (3)	0.083 (4)	−0.018 (3)	0.024 (3)	−0.012 (3)
C64	0.036 (3)	0.078 (4)	0.062 (4)	−0.015 (3)	0.011 (3)	−0.007 (3)
C65	0.034 (3)	0.069 (4)	0.067 (4)	0.007 (3)	0.010 (3)	0.008 (3)
C66	0.038 (3)	0.045 (3)	0.053 (3)	−0.003 (2)	0.008 (2)	0.005 (2)
C71	0.032 (2)	0.029 (2)	0.043 (3)	0.003 (2)	0.013 (2)	0.010 (2)
C72	0.054 (3)	0.038 (3)	0.061 (3)	−0.010 (3)	0.016 (3)	0.009 (3)
C73	0.062 (4)	0.050 (4)	0.104 (5)	−0.019 (3)	0.022 (4)	0.014 (4)
C74	0.055 (4)	0.060 (4)	0.110 (6)	0.002 (3)	0.034 (4)	0.048 (4)
C75	0.063 (4)	0.078 (4)	0.063 (4)	0.015 (3)	0.026 (3)	0.040 (3)
C76	0.040 (3)	0.050 (3)	0.047 (3)	0.002 (2)	0.015 (2)	0.016 (2)
C81	0.029 (2)	0.024 (2)	0.037 (2)	−0.0024 (18)	0.0088 (19)	−0.0043 (19)
C82	0.042 (3)	0.040 (3)	0.043 (3)	0.010 (2)	0.014 (2)	0.001 (2)
C83	0.044 (3)	0.047 (3)	0.057 (3)	0.011 (2)	0.006 (3)	−0.001 (3)
C84	0.037 (3)	0.053 (3)	0.070 (4)	0.005 (3)	0.018 (3)	−0.012 (3)
C85	0.063 (4)	0.066 (4)	0.050 (3)	0.009 (3)	0.030 (3)	−0.005 (3)
C86	0.044 (3)	0.047 (3)	0.039 (3)	0.008 (2)	0.010 (2)	0.000 (2)
C98	0.075 (4)	0.052 (3)	0.055 (3)	−0.010 (3)	0.004 (3)	0.013 (3)
C99	0.041 (3)	0.071 (4)	0.085 (4)	0.003 (3)	0.030 (3)	0.012 (3)

### Geometric parameters (Å, °)

**Table:**

Sb1—O121	1.958 (3)	C33—H33	0.9500
Sb1—O411	1.965 (3)	C34—C35	1.363 (9)
Sb1—C21	2.114 (4)	C34—H34	0.9500
Sb1—C11	2.116 (4)	C35—C36	1.370 (7)
Sb1—O412	2.211 (3)	C35—H35	0.9500
Sb1—O122	2.255 (3)	C36—H36	0.9500
Sb1—Sb2	3.2422 (4)	C41—C46	1.375 (6)
Sb1—Sb4	3.2519 (4)	C41—C42	1.389 (6)
Sb2—O121	1.966 (3)	C42—C43	1.393 (7)
Sb2—O231	1.971 (3)	C42—H42	0.9500
Sb2—C31	2.105 (4)	C43—C44	1.367 (8)
Sb2—C41	2.124 (4)	C43—H43	0.9500
Sb2—O232	2.219 (3)	C44—C45	1.377 (8)
Sb2—O122	2.233 (3)	C44—H44	0.9500
Sb2—Sb3	3.2518 (4)	C45—C46	1.388 (7)
Sb3—O341	1.956 (3)	C45—H45	0.9500
Sb3—O231	1.967 (3)	C46—H46	0.9500
Sb3—C51	2.108 (4)	C51—C52	1.346 (7)
Sb3—C61	2.120 (4)	C51—C56	1.374 (6)
Sb3—O232	2.233 (3)	C52—C53	1.394 (7)
Sb3—O342	2.243 (3)	C52—H52	0.9500
Sb3—Sb4	3.2635 (4)	C53—C54	1.361 (8)
Sb4—O341	1.956 (3)	C53—H53	0.9500
Sb4—O411	1.958 (3)	C54—C55	1.349 (8)
Sb4—C81	2.110 (4)	C54—H54	0.9500
Sb4—C71	2.115 (4)	C55—C56	1.387 (7)
Sb4—O412	2.242 (3)	C55—H55	0.9500
Sb4—O342	2.248 (3)	C56—H56	0.9500
Cl94—C98	1.712 (6)	C61—C62	1.380 (6)
Cl95—C98	1.734 (6)	C61—C66	1.387 (6)
Cl96—C98	1.724 (7)	C62—C63	1.388 (7)
Cl97—C99	1.708 (6)	C62—H62	0.9500
Cl98—C99	1.732 (6)	C63—C64	1.361 (7)
Cl99—C99	1.741 (6)	C63—H63	0.9500
O122—O342	1.473 (3)	C64—C65	1.369 (7)
O232—O412	1.466 (3)	C64—H64	0.9500
C11—C12	1.385 (6)	C65—C66	1.380 (7)
C11—C16	1.389 (6)	C65—H65	0.9500
C12—C13	1.391 (6)	C66—H66	0.9500
C12—H12	0.9500	C71—C76	1.373 (6)
C13—C14	1.367 (7)	C71—C72	1.375 (6)
C13—H13	0.9500	C72—C73	1.385 (7)
C14—C15	1.383 (7)	C72—H72	0.9500
C14—H14	0.9500	C73—C74	1.352 (8)
C15—C16	1.387 (6)	C73—H73	0.9500
C15—H15	0.9500	C74—C75	1.363 (8)
C16—H16	0.9500	C74—H74	0.9500
C21—C22	1.379 (6)	C75—C76	1.386 (7)
C21—C26	1.385 (6)	C75—H75	0.9500
C22—C23	1.386 (6)	C76—H76	0.9500
C22—H22	0.9500	C81—C86	1.378 (6)
C23—C24	1.375 (7)	C81—C82	1.383 (6)
C23—H23	0.9500	C82—C83	1.376 (6)
C24—C25	1.376 (7)	C82—H82	0.9500
C24—H24	0.9500	C83—C84	1.371 (7)
C25—C26	1.378 (6)	C83—H83	0.9500
C25—H25	0.9500	C84—C85	1.377 (7)
C26—H26	0.9500	C84—H84	0.9500
C31—C32	1.371 (6)	C85—C86	1.385 (7)
C31—C36	1.371 (6)	C85—H85	0.9500
C32—C33	1.395 (7)	C86—H86	0.9500
C32—H32	0.9500	C98—H98	1.0000
C33—C34	1.358 (8)	C99—H99	1.0000
			
O121—Sb1—O411	154.46 (11)	C16—C15—H15	119.9
O121—Sb1—C21	95.97 (14)	C15—C16—C11	119.8 (4)
O411—Sb1—C21	98.61 (14)	C15—C16—H16	120.1
O121—Sb1—C11	100.52 (14)	C11—C16—H16	120.1
O411—Sb1—C11	95.73 (14)	C22—C21—C26	119.2 (4)
C21—Sb1—C11	105.26 (15)	C22—C21—Sb1	121.6 (3)
O121—Sb1—O412	84.66 (10)	C26—C21—Sb1	119.2 (3)
O411—Sb1—O412	74.09 (10)	C21—C22—C23	120.2 (4)
C21—Sb1—O412	91.66 (13)	C21—C22—H22	119.9
C11—Sb1—O412	161.55 (13)	C23—C22—H22	119.9
O121—Sb1—O122	74.82 (10)	C24—C23—C22	120.4 (5)
O411—Sb1—O122	85.80 (10)	C24—C23—H23	119.8
C21—Sb1—O122	163.92 (13)	C22—C23—H23	119.8
C11—Sb1—O122	89.57 (13)	C23—C24—C25	119.6 (5)
O412—Sb1—O122	74.60 (10)	C23—C24—H24	120.2
O121—Sb1—Sb2	34.36 (7)	C25—C24—H24	120.2
O411—Sb1—Sb2	121.20 (8)	C24—C25—C26	120.4 (5)
C21—Sb1—Sb2	123.39 (11)	C24—C25—H25	119.8
C11—Sb1—Sb2	108.46 (11)	C26—C25—H25	119.8
O412—Sb1—Sb2	66.34 (7)	C25—C26—C21	120.3 (5)
O122—Sb1—Sb2	43.49 (7)	C25—C26—H26	119.8
O121—Sb1—Sb4	120.93 (7)	C21—C26—H26	119.8
O411—Sb1—Sb4	33.94 (8)	C32—C31—C36	118.1 (5)
C21—Sb1—Sb4	108.85 (11)	C32—C31—Sb2	122.5 (3)
C11—Sb1—Sb4	121.56 (11)	C36—C31—Sb2	119.4 (4)
O412—Sb1—Sb4	43.47 (7)	C31—C32—C33	120.4 (5)
O122—Sb1—Sb4	66.84 (6)	C31—C32—H32	119.8
Sb2—Sb1—Sb4	90.199 (9)	C33—C32—H32	119.8
O121—Sb2—O231	154.00 (11)	C34—C33—C32	120.3 (6)
O121—Sb2—C31	97.76 (14)	C34—C33—H33	119.9
O231—Sb2—C31	98.27 (15)	C32—C33—H33	119.9
O121—Sb2—C41	96.92 (14)	C33—C34—C35	119.7 (6)
O231—Sb2—C41	98.87 (14)	C33—C34—H34	120.2
C31—Sb2—C41	104.04 (17)	C35—C34—H34	120.2
O121—Sb2—O232	84.49 (10)	C34—C35—C36	120.0 (6)
O231—Sb2—O232	74.92 (10)	C34—C35—H35	120.0
C31—Sb2—O232	165.00 (14)	C36—C35—H35	120.0
C41—Sb2—O232	90.34 (14)	C35—C36—C31	121.7 (6)
O121—Sb2—O122	75.17 (10)	C35—C36—H36	119.2
O231—Sb2—O122	84.15 (10)	C31—C36—H36	119.2
C31—Sb2—O122	91.22 (13)	C46—C41—C42	120.6 (4)
C41—Sb2—O122	163.76 (15)	C46—C41—Sb2	120.4 (3)
O232—Sb2—O122	74.96 (10)	C42—C41—Sb2	119.0 (4)
O121—Sb2—Sb1	34.19 (8)	C41—C42—C43	119.5 (5)
O231—Sb2—Sb1	120.56 (8)	C41—C42—H42	120.3
C31—Sb2—Sb1	107.65 (11)	C43—C42—H42	120.3
C41—Sb2—Sb1	123.68 (13)	C44—C43—C42	119.9 (5)
O232—Sb2—Sb1	66.38 (6)	C44—C43—H43	120.0
O122—Sb2—Sb1	44.01 (7)	C42—C43—H43	120.0
O121—Sb2—Sb3	120.62 (8)	C43—C44—C45	120.4 (5)
O231—Sb2—Sb3	34.31 (8)	C43—C44—H44	119.8
C31—Sb2—Sb3	125.92 (12)	C45—C44—H44	119.8
C41—Sb2—Sb3	107.03 (11)	C44—C45—C46	120.6 (5)
O232—Sb2—Sb3	43.24 (7)	C44—C45—H45	119.7
O122—Sb2—Sb3	66.87 (6)	C46—C45—H45	119.7
Sb1—Sb2—Sb3	90.174 (9)	C41—C46—C45	119.2 (5)
O341—Sb3—O231	153.51 (11)	C41—C46—H46	120.4
O341—Sb3—C51	98.58 (14)	C45—C46—H46	120.4
O231—Sb3—C51	95.96 (14)	C52—C51—C56	118.8 (5)
O341—Sb3—C61	95.88 (15)	C52—C51—Sb3	122.9 (3)
O231—Sb3—C61	101.72 (15)	C56—C51—Sb3	118.3 (4)
C51—Sb3—C61	104.61 (16)	C51—C52—C53	121.3 (5)
O341—Sb3—O232	86.57 (10)	C51—C52—H52	119.3
O231—Sb3—O232	74.67 (10)	C53—C52—H52	119.3
C51—Sb3—O232	166.08 (14)	C54—C53—C52	119.5 (6)
C61—Sb3—O232	87.59 (13)	C54—C53—H53	120.3
O341—Sb3—O342	73.03 (10)	C52—C53—H53	120.3
O231—Sb3—O342	83.82 (10)	C55—C54—C53	119.7 (5)
C51—Sb3—O342	94.77 (14)	C55—C54—H54	120.2
C61—Sb3—O342	159.04 (13)	C53—C54—H54	120.2
O232—Sb3—O342	74.27 (9)	C54—C55—C56	120.8 (5)
O341—Sb3—Sb2	120.97 (7)	C54—C55—H55	119.6
O231—Sb3—Sb2	34.38 (8)	C56—C55—H55	119.6
C51—Sb3—Sb2	124.99 (12)	C51—C56—C55	120.0 (5)
C61—Sb3—Sb2	107.22 (11)	C51—C56—H56	120.0
O232—Sb3—Sb2	42.92 (7)	C55—C56—H56	120.0
O342—Sb3—Sb2	66.30 (6)	C62—C61—C66	119.8 (4)
O341—Sb3—Sb4	33.45 (7)	C62—C61—Sb3	121.7 (3)
O231—Sb3—Sb4	120.15 (8)	C66—C61—Sb3	118.5 (3)
C51—Sb3—Sb4	111.44 (12)	C61—C62—C63	119.3 (5)
C61—Sb3—Sb4	119.53 (12)	C61—C62—H62	120.4
O232—Sb3—Sb4	66.54 (7)	C63—C62—H62	120.4
O342—Sb3—Sb4	43.46 (7)	C64—C63—C62	120.8 (5)
Sb2—Sb3—Sb4	89.826 (9)	C64—C63—H63	119.6
O341—Sb4—O411	154.13 (11)	C62—C63—H63	119.6
O341—Sb4—C81	92.93 (14)	C63—C64—C65	120.1 (5)
O411—Sb4—C81	102.85 (14)	C63—C64—H64	119.9
O341—Sb4—C71	99.71 (15)	C65—C64—H64	119.9
O411—Sb4—C71	95.84 (14)	C64—C65—C66	120.3 (5)
C81—Sb4—C71	105.11 (16)	C64—C65—H65	119.9
O341—Sb4—O412	86.17 (10)	C66—C65—H65	119.9
O411—Sb4—O412	73.51 (10)	C65—C66—C61	119.8 (5)
C81—Sb4—O412	90.38 (13)	C65—C66—H66	120.1
C71—Sb4—O412	163.01 (13)	C61—C66—H66	120.1
O341—Sb4—O342	72.92 (10)	C76—C71—C72	119.1 (4)
O411—Sb4—O342	85.92 (10)	C76—C71—Sb4	122.1 (3)
C81—Sb4—O342	159.23 (13)	C72—C71—Sb4	118.8 (3)
C71—Sb4—O342	92.48 (14)	C71—C72—C73	120.2 (5)
O412—Sb4—O342	73.91 (10)	C71—C72—H72	119.9
O341—Sb4—Sb1	120.84 (8)	C73—C72—H72	119.9
O411—Sb4—Sb1	34.09 (8)	C74—C73—C72	119.9 (6)
C81—Sb4—Sb1	110.75 (11)	C74—C73—H73	120.1
C71—Sb4—Sb1	122.70 (12)	C72—C73—H73	120.1
O412—Sb4—Sb1	42.72 (7)	C73—C74—C75	120.8 (5)
O342—Sb4—Sb1	66.62 (6)	C73—C74—H74	119.6
O341—Sb4—Sb3	33.46 (8)	C75—C74—H74	119.6
O411—Sb4—Sb3	121.06 (8)	C74—C75—C76	119.6 (6)
C81—Sb4—Sb3	117.96 (11)	C74—C75—H75	120.2
C71—Sb4—Sb3	110.90 (12)	C76—C75—H75	120.2
O412—Sb4—Sb3	66.09 (6)	C71—C76—C75	120.3 (5)
O342—Sb4—Sb3	43.34 (7)	C71—C76—H76	119.9
Sb1—Sb4—Sb3	89.797 (9)	C75—C76—H76	119.9
Sb1—O121—Sb2	111.45 (12)	C86—C81—C82	118.9 (4)
O342—O122—Sb2	113.13 (17)	C86—C81—Sb4	125.2 (3)
O342—O122—Sb1	112.80 (18)	C82—C81—Sb4	115.7 (3)
Sb2—O122—Sb1	92.50 (10)	C83—C82—C81	121.3 (5)
Sb3—O231—Sb2	111.31 (13)	C83—C82—H82	119.3
O412—O232—Sb2	113.29 (17)	C81—C82—H82	119.3
O412—O232—Sb3	113.51 (17)	C84—C83—C82	119.4 (5)
Sb2—O232—Sb3	93.84 (10)	C84—C83—H83	120.3
Sb4—O341—Sb3	113.08 (13)	C82—C83—H83	120.3
O122—O342—Sb3	113.69 (18)	C83—C84—C85	120.0 (5)
O122—O342—Sb4	113.73 (18)	C83—C84—H84	120.0
Sb3—O342—Sb4	93.20 (10)	C85—C84—H84	120.0
Sb4—O411—Sb1	111.97 (13)	C84—C85—C86	120.4 (5)
O232—O412—Sb1	113.98 (18)	C84—C85—H85	119.8
O232—O412—Sb4	113.86 (17)	C86—C85—H85	119.8
Sb1—O412—Sb4	93.82 (10)	C81—C86—C85	119.9 (5)
C12—C11—C16	119.4 (4)	C81—C86—H86	120.0
C12—C11—Sb1	120.1 (3)	C85—C86—H86	120.0
C16—C11—Sb1	120.4 (3)	Cl94—C98—Cl96	111.9 (4)
C11—C12—C13	120.3 (4)	Cl94—C98—Cl95	109.9 (3)
C11—C12—H12	119.8	Cl96—C98—Cl95	109.5 (3)
C13—C12—H12	119.8	Cl94—C98—H98	108.5
C14—C13—C12	120.0 (5)	Cl96—C98—H98	108.5
C14—C13—H13	120.0	Cl95—C98—H98	108.5
C12—C13—H13	120.0	Cl97—C99—Cl98	110.9 (3)
C13—C14—C15	120.1 (4)	Cl97—C99—Cl99	111.3 (4)
C13—C14—H14	119.9	Cl98—C99—Cl99	109.3 (3)
C15—C14—H14	119.9	Cl97—C99—H99	108.4
C14—C15—C16	120.3 (4)	Cl98—C99—H99	108.4
C14—C15—H15	119.9	Cl99—C99—H99	108.4
			
O411—Sb1—Sb2—O121	169.48 (17)	O342—Sb3—O232—Sb2	71.92 (10)
C21—Sb1—Sb2—O121	41.98 (19)	Sb4—Sb3—O232—Sb2	117.50 (9)
C11—Sb1—Sb2—O121	−81.54 (19)	O411—Sb4—O341—Sb3	12.7 (4)
O412—Sb1—Sb2—O121	117.44 (16)	C81—Sb4—O341—Sb3	140.72 (17)
O122—Sb1—Sb2—O121	−150.23 (18)	C71—Sb4—O341—Sb3	−113.41 (17)
Sb4—Sb1—Sb2—O121	154.89 (15)	O412—Sb4—O341—Sb3	50.53 (14)
O121—Sb1—Sb2—O231	−171.18 (17)	O342—Sb4—O341—Sb3	−23.80 (13)
O411—Sb1—Sb2—O231	−1.70 (14)	Sb1—Sb4—O341—Sb3	24.37 (18)
C21—Sb1—Sb2—O231	−129.20 (16)	O231—Sb3—O341—Sb4	−6.3 (3)
C11—Sb1—Sb2—O231	107.27 (15)	C51—Sb3—O341—Sb4	116.23 (17)
O412—Sb1—Sb2—O231	−53.74 (12)	C61—Sb3—O341—Sb4	−137.98 (16)
O122—Sb1—Sb2—O231	38.59 (13)	O232—Sb3—O341—Sb4	−50.76 (14)
Sb4—Sb1—Sb2—O231	−16.29 (10)	O342—Sb3—O341—Sb4	23.84 (13)
O121—Sb1—Sb2—C31	77.52 (19)	Sb2—Sb3—O341—Sb4	−23.84 (18)
O411—Sb1—Sb2—C31	−113.00 (16)	Sb2—O122—O342—Sb3	0.6 (2)
C21—Sb1—Sb2—C31	119.50 (18)	Sb1—O122—O342—Sb3	103.95 (16)
C11—Sb1—Sb2—C31	−4.02 (17)	Sb2—O122—O342—Sb4	−104.45 (16)
O412—Sb1—Sb2—C31	−165.04 (15)	Sb1—O122—O342—Sb4	−1.1 (2)
O122—Sb1—Sb2—C31	−72.71 (16)	O341—Sb3—O342—O122	−136.6 (2)
Sb4—Sb1—Sb2—C31	−127.59 (13)	O231—Sb3—O342—O122	30.4 (2)
O121—Sb1—Sb2—C41	−43.7 (2)	C51—Sb3—O342—O122	125.9 (2)
O411—Sb1—Sb2—C41	125.80 (17)	C61—Sb3—O342—O122	−76.4 (4)
C21—Sb1—Sb2—C41	−1.70 (19)	O232—Sb3—O342—O122	−45.39 (19)
C11—Sb1—Sb2—C41	−125.22 (18)	Sb2—Sb3—O342—O122	−0.41 (17)
O412—Sb1—Sb2—C41	73.76 (16)	Sb4—Sb3—O342—O122	−117.7 (2)
O122—Sb1—Sb2—C41	166.09 (17)	O341—Sb3—O342—Sb4	−18.90 (10)
Sb4—Sb1—Sb2—C41	111.21 (14)	O231—Sb3—O342—Sb4	148.07 (11)
O121—Sb1—Sb2—O232	−117.20 (16)	C51—Sb3—O342—Sb4	−116.43 (14)
O411—Sb1—Sb2—O232	52.28 (12)	C61—Sb3—O342—Sb4	41.3 (4)
C21—Sb1—Sb2—O232	−75.22 (15)	O232—Sb3—O342—Sb4	72.30 (10)
C11—Sb1—Sb2—O232	161.26 (14)	Sb2—Sb3—O342—Sb4	117.28 (9)
O412—Sb1—Sb2—O232	0.24 (10)	O341—Sb4—O342—O122	136.6 (2)
O122—Sb1—Sb2—O232	92.57 (12)	O411—Sb4—O342—O122	−28.3 (2)
Sb4—Sb1—Sb2—O232	37.69 (7)	C81—Sb4—O342—O122	87.8 (4)
O121—Sb1—Sb2—O122	150.23 (18)	C71—Sb4—O342—O122	−124.0 (2)
O411—Sb1—Sb2—O122	−40.29 (14)	O412—Sb4—O342—O122	45.65 (18)
C21—Sb1—Sb2—O122	−167.79 (16)	Sb1—Sb4—O342—O122	0.76 (16)
C11—Sb1—Sb2—O122	68.68 (15)	Sb3—Sb4—O342—O122	117.7 (2)
O412—Sb1—Sb2—O122	−92.33 (12)	O341—Sb4—O342—Sb3	18.91 (10)
Sb4—Sb1—Sb2—O122	−54.88 (10)	O411—Sb4—O342—Sb3	−146.00 (11)
O121—Sb1—Sb2—Sb3	−154.41 (15)	C81—Sb4—O342—Sb3	−29.8 (4)
O411—Sb1—Sb2—Sb3	15.07 (10)	C71—Sb4—O342—Sb3	118.32 (14)
C21—Sb1—Sb2—Sb3	−112.43 (13)	O412—Sb4—O342—Sb3	−72.00 (9)
C11—Sb1—Sb2—Sb3	124.04 (12)	Sb1—Sb4—O342—Sb3	−116.90 (9)
O412—Sb1—Sb2—Sb3	−36.97 (7)	O341—Sb4—O411—Sb1	18.1 (4)
O122—Sb1—Sb2—Sb3	55.36 (10)	C81—Sb4—O411—Sb1	−108.14 (16)
Sb4—Sb1—Sb2—Sb3	0.478 (10)	C71—Sb4—O411—Sb1	144.89 (17)
O121—Sb2—Sb3—O341	−3.98 (14)	O412—Sb4—O411—Sb1	−21.60 (12)
O231—Sb2—Sb3—O341	166.25 (18)	O342—Sb4—O411—Sb1	52.81 (14)
C31—Sb2—Sb3—O341	124.52 (17)	Sb3—Sb4—O411—Sb1	26.19 (17)
C41—Sb2—Sb3—O341	−113.12 (17)	O121—Sb1—O411—Sb4	−13.0 (4)
O232—Sb2—Sb3—O341	−41.58 (14)	C21—Sb1—O411—Sb4	111.12 (16)
O122—Sb2—Sb3—O341	50.83 (13)	C11—Sb1—O411—Sb4	−142.46 (16)
Sb1—Sb2—Sb3—O341	12.40 (10)	O412—Sb1—O411—Sb4	21.86 (12)
O121—Sb2—Sb3—O231	−170.23 (17)	O122—Sb1—O411—Sb4	−53.32 (14)
C31—Sb2—Sb3—O231	−41.7 (2)	Sb2—Sb1—O411—Sb4	−26.81 (17)
C41—Sb2—Sb3—O231	80.6 (2)	Sb2—O232—O412—Sb1	0.5 (2)
O232—Sb2—Sb3—O231	152.17 (18)	Sb3—O232—O412—Sb1	106.01 (16)
O122—Sb2—Sb3—O231	−115.42 (17)	Sb2—O232—O412—Sb4	−105.51 (16)
Sb1—Sb2—Sb3—O231	−153.85 (15)	Sb3—O232—O412—Sb4	0.0 (2)
O121—Sb2—Sb3—C51	−133.22 (17)	O121—Sb1—O412—O232	29.83 (19)
O231—Sb2—Sb3—C51	37.0 (2)	O411—Sb1—O412—O232	−135.8 (2)
C31—Sb2—Sb3—C51	−4.7 (2)	C21—Sb1—O412—O232	125.7 (2)
C41—Sb2—Sb3—C51	117.6 (2)	C11—Sb1—O412—O232	−77.6 (5)
O232—Sb2—Sb3—C51	−170.82 (17)	O122—Sb1—O412—O232	−45.87 (19)
O122—Sb2—Sb3—C51	−78.40 (16)	Sb2—Sb1—O412—O232	−0.37 (16)
Sb1—Sb2—Sb3—C51	−116.83 (14)	Sb4—Sb1—O412—O232	−118.3 (2)
O121—Sb2—Sb3—C61	104.15 (15)	O121—Sb1—O412—Sb4	148.09 (11)
O231—Sb2—Sb3—C61	−85.62 (19)	O411—Sb1—O412—Sb4	−17.59 (10)
C31—Sb2—Sb3—C61	−127.34 (18)	C21—Sb1—O412—Sb4	−116.07 (13)
C41—Sb2—Sb3—C61	−4.99 (18)	C11—Sb1—O412—Sb4	40.7 (4)
O232—Sb2—Sb3—C61	66.55 (15)	O122—Sb1—O412—Sb4	72.39 (10)
O122—Sb2—Sb3—C61	158.96 (14)	Sb2—Sb1—O412—Sb4	117.89 (9)
Sb1—Sb2—Sb3—C61	120.53 (12)	O341—Sb4—O412—O232	−27.7 (2)
O121—Sb2—Sb3—O232	37.60 (14)	O411—Sb4—O412—O232	136.1 (2)
O231—Sb2—Sb3—O232	−152.17 (17)	C81—Sb4—O412—O232	−120.6 (2)
C31—Sb2—Sb3—O232	166.11 (17)	C71—Sb4—O412—O232	83.4 (5)
C41—Sb2—Sb3—O232	−71.54 (17)	O342—Sb4—O412—O232	45.59 (19)
O122—Sb2—Sb3—O232	92.41 (12)	Sb1—Sb4—O412—O232	118.4 (2)
Sb1—Sb2—Sb3—O232	53.98 (10)	Sb3—Sb4—O412—O232	0.03 (16)
O121—Sb2—Sb3—O342	−54.54 (12)	O341—Sb4—O412—Sb1	−146.08 (11)
O231—Sb2—Sb3—O342	115.69 (17)	O411—Sb4—O412—Sb1	17.71 (10)
C31—Sb2—Sb3—O342	73.96 (16)	C81—Sb4—O412—Sb1	121.00 (13)
C41—Sb2—Sb3—O342	−163.68 (16)	C71—Sb4—O412—Sb1	−35.0 (5)
O232—Sb2—Sb3—O342	−92.14 (12)	O342—Sb4—O412—Sb1	−72.76 (10)
O122—Sb2—Sb3—O342	0.27 (11)	Sb3—Sb4—O412—Sb1	−118.33 (9)
Sb1—Sb2—Sb3—O342	−38.16 (8)	O121—Sb1—C11—C12	−174.4 (3)
O121—Sb2—Sb3—Sb4	−16.86 (10)	O411—Sb1—C11—C12	−14.2 (4)
O231—Sb2—Sb3—Sb4	153.37 (15)	C21—Sb1—C11—C12	86.4 (4)
C31—Sb2—Sb3—Sb4	111.65 (14)	O412—Sb1—C11—C12	−69.4 (6)
C41—Sb2—Sb3—Sb4	−126.00 (14)	O122—Sb1—C11—C12	−99.9 (3)
O232—Sb2—Sb3—Sb4	−54.46 (10)	Sb2—Sb1—C11—C12	−139.8 (3)
O122—Sb2—Sb3—Sb4	37.95 (8)	Sb4—Sb1—C11—C12	−37.7 (4)
Sb1—Sb2—Sb3—Sb4	−0.477 (10)	O121—Sb1—C11—C16	9.2 (4)
O121—Sb1—Sb4—O341	2.58 (14)	O411—Sb1—C11—C16	169.4 (3)
O411—Sb1—Sb4—O341	−170.93 (18)	C21—Sb1—C11—C16	−90.0 (4)
C21—Sb1—Sb4—O341	112.01 (15)	O412—Sb1—C11—C16	114.1 (4)
C11—Sb1—Sb4—O341	−125.58 (16)	O122—Sb1—C11—C16	83.7 (3)
O412—Sb1—Sb4—O341	40.42 (14)	Sb2—Sb1—C11—C16	43.8 (4)
O122—Sb1—Sb4—O341	−51.38 (12)	Sb4—Sb1—C11—C16	145.9 (3)
Sb2—Sb1—Sb4—O341	−13.63 (10)	C16—C11—C12—C13	0.3 (7)
O121—Sb1—Sb4—O411	173.52 (18)	Sb1—C11—C12—C13	−176.2 (4)
C21—Sb1—Sb4—O411	−77.05 (19)	C11—C12—C13—C14	−0.9 (7)
C11—Sb1—Sb4—O411	45.4 (2)	C12—C13—C14—C15	0.9 (8)
O412—Sb1—Sb4—O411	−148.64 (18)	C13—C14—C15—C16	−0.2 (7)
O122—Sb1—Sb4—O411	119.55 (17)	C14—C15—C16—C11	−0.4 (7)
Sb2—Sb1—Sb4—O411	157.31 (15)	C12—C11—C16—C15	0.3 (7)
O121—Sb1—Sb4—C81	−104.27 (15)	Sb1—C11—C16—C15	176.8 (3)
O411—Sb1—Sb4—C81	82.21 (19)	O121—Sb1—C21—C22	40.7 (4)
C21—Sb1—Sb4—C81	5.16 (17)	O411—Sb1—C21—C22	−118.3 (4)
C11—Sb1—Sb4—C81	127.57 (17)	C11—Sb1—C21—C22	143.3 (4)
O412—Sb1—Sb4—C81	−66.43 (15)	O412—Sb1—C21—C22	−44.1 (4)
O122—Sb1—Sb4—C81	−158.24 (14)	O122—Sb1—C21—C22	−13.3 (7)
Sb2—Sb1—Sb4—C81	−120.48 (11)	Sb2—Sb1—C21—C22	18.4 (4)
O121—Sb1—Sb4—C71	130.67 (18)	Sb4—Sb1—C21—C22	−84.9 (4)
O411—Sb1—Sb4—C71	−42.8 (2)	O121—Sb1—C21—C26	−137.7 (3)
C21—Sb1—Sb4—C71	−119.90 (19)	O411—Sb1—C21—C26	63.3 (3)
C11—Sb1—Sb4—C71	2.5 (2)	C11—Sb1—C21—C26	−35.1 (4)
O412—Sb1—Sb4—C71	168.51 (18)	O412—Sb1—C21—C26	137.5 (3)
O122—Sb1—Sb4—C71	76.70 (16)	O122—Sb1—C21—C26	168.3 (3)
Sb2—Sb1—Sb4—C71	114.46 (15)	Sb2—Sb1—C21—C26	−160.0 (3)
O121—Sb1—Sb4—O412	−37.84 (14)	Sb4—Sb1—C21—C26	96.7 (3)
O411—Sb1—Sb4—O412	148.64 (18)	C26—C21—C22—C23	−0.2 (7)
C21—Sb1—Sb4—O412	71.59 (15)	Sb1—C21—C22—C23	−178.6 (3)
C11—Sb1—Sb4—O412	−166.00 (16)	C21—C22—C23—C24	−0.6 (7)
O122—Sb1—Sb4—O412	−91.81 (12)	C22—C23—C24—C25	0.9 (8)
Sb2—Sb1—Sb4—O412	−54.05 (10)	C23—C24—C25—C26	−0.4 (8)
O121—Sb1—Sb4—O342	53.47 (12)	C24—C25—C26—C21	−0.4 (8)
O411—Sb1—Sb4—O342	−120.04 (17)	C22—C21—C26—C25	0.7 (7)
C21—Sb1—Sb4—O342	162.91 (14)	Sb1—C21—C26—C25	179.1 (4)
C11—Sb1—Sb4—O342	−74.68 (15)	O121—Sb2—C31—C32	140.5 (4)
O412—Sb1—Sb4—O342	91.32 (12)	O231—Sb2—C31—C32	−19.0 (4)
O122—Sb1—Sb4—O342	−0.49 (10)	C41—Sb2—C31—C32	−120.4 (4)
Sb2—Sb1—Sb4—O342	37.26 (7)	O232—Sb2—C31—C32	42.7 (8)
O121—Sb1—Sb4—Sb3	15.73 (10)	O122—Sb2—C31—C32	65.3 (4)
O411—Sb1—Sb4—Sb3	−157.78 (15)	Sb1—Sb2—C31—C32	106.8 (4)
C21—Sb1—Sb4—Sb3	125.17 (12)	Sb3—Sb2—C31—C32	3.3 (4)
C11—Sb1—Sb4—Sb3	−112.42 (13)	O121—Sb2—C31—C36	−41.7 (4)
O412—Sb1—Sb4—Sb3	53.58 (10)	O231—Sb2—C31—C36	158.9 (4)
O122—Sb1—Sb4—Sb3	−38.23 (7)	C41—Sb2—C31—C36	57.5 (4)
Sb2—Sb1—Sb4—Sb3	−0.477 (10)	O232—Sb2—C31—C36	−139.4 (5)
O231—Sb3—Sb4—O341	176.74 (18)	O122—Sb2—C31—C36	−116.9 (4)
C51—Sb3—Sb4—O341	−72.3 (2)	Sb1—Sb2—C31—C36	−75.3 (4)
C61—Sb3—Sb4—O341	49.9 (2)	Sb3—Sb2—C31—C36	−178.8 (3)
O232—Sb3—Sb4—O341	122.57 (17)	C36—C31—C32—C33	−0.7 (8)
O342—Sb3—Sb4—O341	−145.81 (18)	Sb2—C31—C32—C33	177.2 (4)
Sb2—Sb3—Sb4—O341	159.72 (15)	C31—C32—C33—C34	−0.5 (9)
O341—Sb3—Sb4—O411	−173.57 (18)	C32—C33—C34—C35	1.5 (9)
O231—Sb3—Sb4—O411	3.17 (13)	C33—C34—C35—C36	−1.3 (10)
C51—Sb3—Sb4—O411	114.09 (16)	C34—C35—C36—C31	0.2 (9)
C61—Sb3—Sb4—O411	−123.64 (16)	C32—C31—C36—C35	0.8 (8)
O232—Sb3—Sb4—O411	−51.00 (12)	Sb2—C31—C36—C35	−177.1 (4)
O342—Sb3—Sb4—O411	40.62 (14)	O121—Sb2—C41—C46	−54.5 (4)
Sb2—Sb3—Sb4—O411	−13.85 (10)	O231—Sb2—C41—C46	104.8 (4)
O341—Sb3—Sb4—C81	−45.7 (2)	C31—Sb2—C41—C46	−154.3 (4)
O231—Sb3—Sb4—C81	131.02 (16)	O232—Sb2—C41—C46	30.0 (4)
C51—Sb3—Sb4—C81	−118.06 (18)	O122—Sb2—C41—C46	5.2 (7)
C61—Sb3—Sb4—C81	4.21 (18)	Sb1—Sb2—C41—C46	−31.4 (4)
O232—Sb3—Sb4—C81	76.85 (15)	Sb3—Sb2—C41—C46	70.6 (4)
O342—Sb3—Sb4—C81	168.47 (16)	O121—Sb2—C41—C42	125.0 (3)
Sb2—Sb3—Sb4—C81	114.00 (13)	O231—Sb2—C41—C42	−75.7 (3)
O341—Sb3—Sb4—C71	75.5 (2)	C31—Sb2—C41—C42	25.2 (4)
O231—Sb3—Sb4—C71	−107.74 (15)	O232—Sb2—C41—C42	−150.5 (3)
C51—Sb3—Sb4—C71	3.18 (18)	O122—Sb2—C41—C42	−175.3 (3)
C61—Sb3—Sb4—C71	125.44 (18)	Sb1—Sb2—C41—C42	148.0 (3)
O232—Sb3—Sb4—C71	−161.92 (14)	Sb3—Sb2—C41—C42	−110.0 (3)
O342—Sb3—Sb4—C71	−70.30 (15)	C46—C41—C42—C43	0.9 (7)
Sb2—Sb3—Sb4—C71	−124.76 (12)	Sb2—C41—C42—C43	−178.5 (3)
O341—Sb3—Sb4—O412	−122.58 (17)	C41—C42—C43—C44	−1.0 (7)
O231—Sb3—Sb4—O412	54.16 (12)	C42—C43—C44—C45	0.1 (8)
C51—Sb3—Sb4—O412	165.08 (15)	C43—C44—C45—C46	0.9 (8)
C61—Sb3—Sb4—O412	−72.65 (15)	C42—C41—C46—C45	0.0 (7)
O232—Sb3—Sb4—O412	−0.02 (10)	Sb2—C41—C46—C45	179.5 (4)
O342—Sb3—Sb4—O412	91.61 (12)	C44—C45—C46—C41	−0.9 (8)
Sb2—Sb3—Sb4—O412	37.14 (7)	O341—Sb3—C51—C52	−85.4 (5)
O341—Sb3—Sb4—O342	145.81 (18)	O231—Sb3—C51—C52	72.4 (5)
O231—Sb3—Sb4—O342	−37.45 (13)	C61—Sb3—C51—C52	176.2 (5)
C51—Sb3—Sb4—O342	73.47 (16)	O232—Sb3—C51—C52	25.6 (9)
C61—Sb3—Sb4—O342	−164.26 (16)	O342—Sb3—C51—C52	−11.8 (5)
O232—Sb3—Sb4—O342	−91.62 (12)	Sb2—Sb3—C51—C52	52.4 (5)
Sb2—Sb3—Sb4—O342	−54.47 (9)	Sb4—Sb3—C51—C52	−53.3 (5)
O341—Sb3—Sb4—Sb1	−159.25 (15)	O341—Sb3—C51—C56	94.3 (4)
O231—Sb3—Sb4—Sb1	17.50 (10)	O231—Sb3—C51—C56	−107.9 (4)
C51—Sb3—Sb4—Sb1	128.42 (13)	C61—Sb3—C51—C56	−4.1 (4)
C61—Sb3—Sb4—Sb1	−109.32 (13)	O232—Sb3—C51—C56	−154.8 (5)
O232—Sb3—Sb4—Sb1	−36.68 (7)	O342—Sb3—C51—C56	167.8 (4)
O342—Sb3—Sb4—Sb1	54.94 (9)	Sb2—Sb3—C51—C56	−127.9 (4)
Sb2—Sb3—Sb4—Sb1	0.475 (10)	Sb4—Sb3—C51—C56	126.4 (4)
O411—Sb1—O121—Sb2	−21.2 (3)	C56—C51—C52—C53	−0.7 (9)
C21—Sb1—O121—Sb2	−145.84 (16)	Sb3—C51—C52—C53	178.9 (5)
C11—Sb1—O121—Sb2	107.39 (16)	C51—C52—C53—C54	1.7 (11)
O412—Sb1—O121—Sb2	−54.73 (13)	C52—C53—C54—C55	−1.9 (11)
O122—Sb1—O121—Sb2	20.74 (12)	C53—C54—C55—C56	1.1 (11)
Sb4—Sb1—O121—Sb2	−29.65 (17)	C52—C51—C56—C55	−0.1 (9)
O231—Sb2—O121—Sb1	17.5 (3)	Sb3—C51—C56—C55	−179.7 (5)
C31—Sb2—O121—Sb1	−110.11 (17)	C54—C55—C56—C51	−0.1 (10)
C41—Sb2—O121—Sb1	144.63 (17)	O341—Sb3—C61—C62	161.9 (4)
O232—Sb2—O121—Sb1	54.95 (13)	O231—Sb3—C61—C62	1.8 (4)
O122—Sb2—O121—Sb1	−20.91 (12)	C51—Sb3—C61—C62	−97.7 (4)
Sb3—Sb2—O121—Sb1	30.12 (17)	O232—Sb3—C61—C62	75.6 (4)
O121—Sb2—O122—O342	132.9 (2)	O342—Sb3—C61—C62	105.3 (5)
O231—Sb2—O122—O342	−31.2 (2)	Sb2—Sb3—C61—C62	36.9 (4)
C31—Sb2—O122—O342	−129.4 (2)	Sb4—Sb3—C61—C62	136.8 (3)
C41—Sb2—O122—O342	70.5 (5)	O341—Sb3—C61—C66	−16.0 (4)
O232—Sb2—O122—O342	44.73 (19)	O231—Sb3—C61—C66	−176.1 (3)
Sb1—Sb2—O122—O342	116.1 (2)	C51—Sb3—C61—C66	84.5 (4)
Sb3—Sb2—O122—O342	−0.41 (17)	O232—Sb3—C61—C66	−102.3 (3)
O121—Sb2—O122—Sb1	16.78 (10)	O342—Sb3—C61—C66	−72.6 (5)
O231—Sb2—O122—Sb1	−147.32 (11)	Sb2—Sb3—C61—C66	−141.0 (3)
C31—Sb2—O122—Sb1	114.49 (14)	Sb4—Sb3—C61—C66	−41.1 (4)
C41—Sb2—O122—Sb1	−45.6 (5)	C66—C61—C62—C63	−0.2 (7)
O232—Sb2—O122—Sb1	−71.40 (10)	Sb3—C61—C62—C63	−178.0 (4)
Sb3—Sb2—O122—Sb1	−116.54 (9)	C61—C62—C63—C64	−0.6 (8)
O121—Sb1—O122—O342	−133.3 (2)	C62—C63—C64—C65	0.5 (9)
O411—Sb1—O122—O342	29.89 (19)	C63—C64—C65—C66	0.3 (9)
C21—Sb1—O122—O342	−76.8 (5)	C64—C65—C66—C61	−1.0 (8)
C11—Sb1—O122—O342	125.7 (2)	C62—C61—C66—C65	1.0 (7)
O412—Sb1—O122—O342	−44.75 (18)	Sb3—C61—C66—C65	178.9 (4)
Sb2—Sb1—O122—O342	−116.4 (2)	O341—Sb4—C71—C76	10.3 (4)
Sb4—Sb1—O122—O342	0.75 (16)	O411—Sb4—C71—C76	−148.9 (4)
O121—Sb1—O122—Sb2	−16.88 (10)	C81—Sb4—C71—C76	106.0 (4)
O411—Sb1—O122—Sb2	146.31 (11)	O412—Sb4—C71—C76	−98.9 (5)
C21—Sb1—O122—Sb2	39.6 (5)	O342—Sb4—C71—C76	−62.8 (4)
C11—Sb1—O122—Sb2	−117.91 (13)	Sb1—Sb4—C71—C76	−126.4 (3)
O412—Sb1—O122—Sb2	71.67 (10)	Sb3—Sb4—C71—C76	−22.5 (4)
Sb4—Sb1—O122—Sb2	117.17 (9)	O341—Sb4—C71—C72	−170.2 (4)
O341—Sb3—O231—Sb2	−27.2 (3)	O411—Sb4—C71—C72	30.5 (4)
C51—Sb3—O231—Sb2	−150.27 (16)	C81—Sb4—C71—C72	−74.5 (4)
C61—Sb3—O231—Sb2	103.43 (16)	O412—Sb4—C71—C72	80.6 (6)
O232—Sb3—O231—Sb2	19.25 (12)	O342—Sb4—C71—C72	116.7 (4)
O342—Sb3—O231—Sb2	−56.10 (13)	Sb1—Sb4—C71—C72	53.0 (4)
Sb4—Sb3—O231—Sb2	−31.22 (16)	Sb3—Sb4—C71—C72	157.0 (3)
O121—Sb2—O231—Sb3	19.5 (3)	C76—C71—C72—C73	−0.1 (8)
C31—Sb2—O231—Sb3	147.00 (16)	Sb4—C71—C72—C73	−179.6 (4)
C41—Sb2—O231—Sb3	−107.29 (18)	C71—C72—C73—C74	1.4 (9)
O232—Sb2—O231—Sb3	−19.34 (12)	C72—C73—C74—C75	−2.1 (10)
O122—Sb2—O231—Sb3	56.61 (14)	C73—C74—C75—C76	1.6 (9)
Sb1—Sb2—O231—Sb3	30.79 (17)	C72—C71—C76—C75	−0.4 (7)
O121—Sb2—O232—O412	−30.51 (19)	Sb4—C71—C76—C75	179.1 (4)
O231—Sb2—O232—O412	133.5 (2)	C74—C75—C76—C71	−0.3 (8)
C31—Sb2—O232—O412	68.9 (6)	O341—Sb4—C81—C86	−126.2 (4)
C41—Sb2—O232—O412	−127.4 (2)	O411—Sb4—C81—C86	33.1 (4)
O122—Sb2—O232—O412	45.58 (18)	C71—Sb4—C81—C86	132.9 (4)
Sb1—Sb2—O232—O412	−0.37 (16)	O412—Sb4—C81—C86	−40.0 (4)
Sb3—Sb2—O232—O412	117.7 (2)	O342—Sb4—C81—C86	−80.2 (5)
O121—Sb2—O232—Sb3	−148.16 (11)	Sb1—Sb4—C81—C86	−1.6 (4)
O231—Sb2—O232—Sb3	15.82 (10)	Sb3—Sb4—C81—C86	−102.9 (4)
C31—Sb2—O232—Sb3	−48.7 (5)	O341—Sb4—C81—C82	48.1 (3)
C41—Sb2—O232—Sb3	114.91 (13)	O411—Sb4—C81—C82	−152.6 (3)
O122—Sb2—O232—Sb3	−72.07 (10)	C71—Sb4—C81—C82	−52.8 (4)
Sb1—Sb2—O232—Sb3	−118.02 (9)	O412—Sb4—C81—C82	134.3 (3)
O341—Sb3—O232—O412	27.8 (2)	O342—Sb4—C81—C82	94.1 (5)
O231—Sb3—O232—O412	−133.3 (2)	Sb1—Sb4—C81—C82	172.7 (3)
C51—Sb3—O232—O412	−84.5 (6)	Sb3—Sb4—C81—C82	71.3 (3)
C61—Sb3—O232—O412	123.8 (2)	C86—C81—C82—C83	−0.1 (7)
O342—Sb3—O232—O412	−45.56 (19)	Sb4—C81—C82—C83	−174.7 (4)
Sb2—Sb3—O232—O412	−117.5 (2)	C81—C82—C83—C84	0.7 (8)
Sb4—Sb3—O232—O412	0.03 (16)	C82—C83—C84—C85	−1.2 (8)
O341—Sb3—O232—Sb2	145.24 (11)	C83—C84—C85—C86	1.1 (8)
O231—Sb3—O232—Sb2	−15.86 (10)	C82—C81—C86—C85	0.0 (7)
C51—Sb3—O232—Sb2	32.9 (6)	Sb4—C81—C86—C85	174.1 (4)
C61—Sb3—O232—Sb2	−118.71 (14)	C84—C85—C86—C81	−0.5 (8)

### Hydrogen-bond geometry (Å, °)

**Table:**

*D*—H···*A*	*D*—H	H···*A*	*D*···*A*	*D*—H···*A*
C74—H74···Cl95^i^	0.95	2.81	3.345 (6)	117
C98—H98···O231^i^	1.00	2.29	3.229 (6)	156
C99—H99···O122^ii^	1.00	2.27	3.275 (6)	177
C99—H99···O342^ii^	1.00	2.46	3.343 (6)	147
C23—H23···O412^iii^	0.95	2.60	3.482 (6)	154

Symmetry codes: (i) −*x*+1/2, *y*−1/2, −*z*+1/2; (ii) *x*+1/2, *y*−1/2, *z*; (iii) −*x*+1/2, −*y*+3/2, −*z*.
